# Wearable System Based on Ultra-Thin Parylene C Tattoo Electrodes for EEG Recording

**DOI:** 10.3390/s23020766

**Published:** 2023-01-09

**Authors:** Antonello Mascia, Riccardo Collu, Andrea Spanu, Matteo Fraschini, Massimo Barbaro, Piero Cosseddu

**Affiliations:** 1 Department of Electrical and Electronics Engineering, University of Cagliari, Piazza D’Armi, 09123 Cagliari, Italy; 2 Department of Science, Technology and Society, Scuola Universitaria Superiore IUSS, Palazzo del Broletto, Piazza della Vittoria 15, 27100 Pavia, Italy

**Keywords:** Parylene C, dry tattoo electrodes, wearable electronic system, EEG

## Abstract

In an increasingly interconnected world, where electronic devices permeate every aspect of our lives, wearable systems aimed at monitoring physiological signals are rapidly taking over the sport and fitness domain, as well as biomedical fields such as rehabilitation and prosthetics. With the intent of providing a novel approach to the field, in this paper we discuss the development of a wearable system for the acquisition of EEG signals based on a portable, low-power custom PCB specifically designed to be used in combination with non-conventional ultra-conformable and imperceptible Parylene-C tattoo electrodes. The proposed system has been tested in a standard rest-state experiment, and its performance in terms of discrimination of two different states has been compared to that of a commercial wearable device for EEG signal acquisition (i.e., the Muse headset), showing comparable results. This first preliminary validation demonstrates the possibility of conveniently employing ultra-conformable tattoo-electrodes integrated portable systems for the unobtrusive acquisition of brain activity.

## 1. Introduction

Being able to monitor physiological parameters using non-invasive methods represents a fundamental requirement in modern biomedical applications, and for this reason, an increasing number of portable and wearable devices are nowadays available for the daily monitoring of different kinds of biopotentials for both clinical and non-clinical applications [1,2,3,4]. Particularly intriguing are all those novel applications involving the non-clinical monitoring of EEG (electroencephalography) signals, applications ranging from human-machine interfaces to innovative approaches for biometry and emotion recognition [5,6,7,8,9,10]. Unfortunately, the recording of brain activity usually requires bulky measurement setups with up to 256 electrodes in high-density systems [11], and it can be carried out only in controlled, often restricted, and uncomfortable environments. With the idea of overcoming these seemingly unavoidable issues, recently, alternative approaches have been proposed, such as the in-ear EEG [12,13,14,15], an interesting solution that allows the size and the encumbrance of the set-up to be reduced, an approach that is, however, limited mainly to the detection of event-related potentials (ERPs). Although at the moment some wearable devices for the acquisition of EEG signals are already present on the market [16,17], their main drawbacks, which are still limiting their widespread employment, are mainly related to the size of the acquisition electronics, the data storage capability, the battery life, and the quality of the employed electrodes. In fact, an ideal electrode for wearable EEG systems should ensure a good signal quality while still being comfortable and, possibly, imperceptible to the user. In this regard, tattoo-electrodes could be an ideal alternative, due to their conformability to the skin [18], which can be achieved without the need for any adhesive layer or conductive gel, substances that are known for their adverse effect on the skin [19,20,21]. The tattoo approach, with different materials and fabrication techniques, has already been extensively explored for many biopotentials ranging from electrocardiography (ECG) recordings [22,23,24] to electromyography (EMG) [25,26,27] and, more recently, EEG [28,29,30,31]. In Table 1, a comparison between different EEG acquisition systems is reported. 

With the intent of providing a new solution that can combine the advantages of the wearable approach to those of epidermal imperceptible electrodes, we present here a wearable acquisition system based on ultrathin Parylene C dry electrodes that can provide high-quality EEG recordings with increased comfort to the user. In fact, Parylene C has recently emerged as a convenient material for the fabrication of tattoo electrodes, thanks to its mechanical properties, biocompatibility, chemical inertness and the possibility of depositing it in the form of ultra-thin, sub-micrometer layers through a reliable and high-throughput chemical vapor deposition technique [22,32]. The proposed system consists of a custom Printed Circuit Board (PCB) fabricated using off-the-shelf components and a modified headband that has been specifically designed to conveniently interface unconventional ultrathin tattoo electrodes. Our approach allows us to drastically reduce the amount of adhesive required for commercial pre-gelled electrodes, and at the same time, provides a unique modular approach that makes it compatible also with different kinds of innovative epidermal solutions [33] by simply replacing the metal contacts with conductive magnets, thus making this approach an interesting candidate for the development of a new generation of wearable systems capable of conveniently and unobtrusively monitoring brain signals.

## 2. Materials and Methods

**Figure 1:**
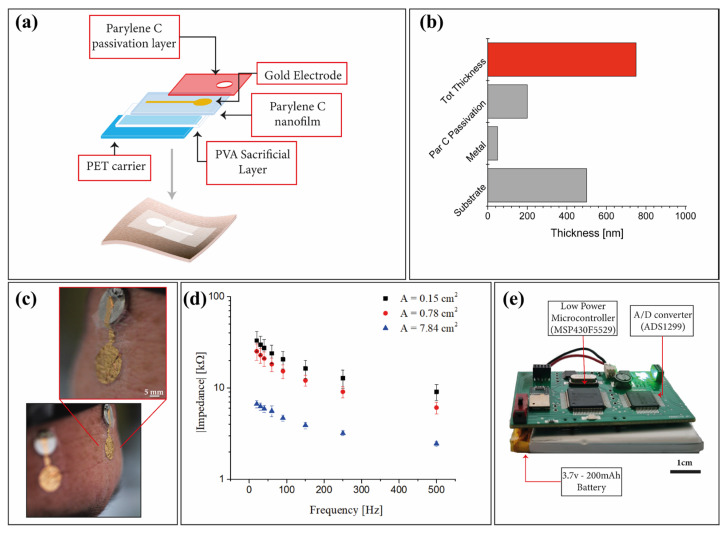
(**a**) Fabrication process flow where all the different layers are highlighted. (**b**) Average thickness of the proposed tattoo-electrodes. (**c**) Ultra-conformable Parylene C tattoo-electrodes applied on the forehead and positioned on a 1 cm metal plate for the signal acquisition. (**d**) Average electrode-skin impedance evaluated for three different contact areas. (**e**) Microcontroller-based custom PCB.

The tattoo-electrodes fabrication process is shown in Figure 1a and starts from a 175 µm-thick poly(ethylene terephthalate) (PET) substrate, in which a poly(vinyl alcohol) (PVA) sacrificial layer is deposited through a standard spin coating technique. After the PVA deposition, a 500 nm Parylene C layer is deposited through chemical vapor deposition (CVD). Afterwards, gold electrodes (50 nm-thick) are deposited onto the Parylene C film by means of thermal evaporation and patterned using a standard photolithographic process. Finally, the electrodes are passivated (with the exception of the recording and the connection areas) with a 200 nm-thick second Parylene C layer. The final electrodes have a thickness of about 800 nm (measured using a stylus profilometer—DektakXT, BRUKER, Madison, WI, USA) as shown in Figure 1b, which ensures a very good conformal interface with the skin thanks only to electrostatic interactions and without using any glue or electrolyte gel, as shown in Figure 1c. In order to be applied onto the skin, the patch is peeled off from the PET carrier and transferred onto a piece of paper using a little amount of deionized water. After the water dries out, the electrode can be stored, and eventually positioned onto the skin by simply wetting the back of the paper using a few droplets of deionized water and gently sliding the paper away. To select the most suitable electrode dimension, and at the same time ensure that the skin-electrode impedance was sufficiently low, as required for electroencephalography [34], three different sets of electrodes with different areas were produced and tested for the electrode-skin impedance. An Agilent 4284A precision LCR meter (Agilent Technologies Inc., Santa Clara, CA, USA) was used to evaluate the impedance of the tattoo electrodes with respect to the parallel of five commercial pre-gelled electrodes in the range of 10–500 Hz, similarly to what has already been reported in [22,33,35]. Interestingly, the obtained impedances are in line with the contact impedance of other types of semi-dry electrodes, as reported in [36]. The results of this test are reported in Figure 1d. To ensure the best comfort while at the same time guaranteeing an optimal skin/electrode impedance, all the electrodes were fabricated with an overall contact area of 0.78 mm^2^.

A custom microcontroller-based PCB with off-the-shelf components (COTS) was developed (Figure 1e) to ensure an efficient signal acquisition. The entire design procedure was performed with the aim of obtaining a low-size and low-power consumption PCB. The brain signals collected by the electrodes are acquired by means of a Texas Instrument 24-bit ΣΔ analog-digital converter ADS1299. Through the ADC, it is possible to turn off the undesired channels (each of which is composed of an instrumentation amplifier with programmable gain between 1 and 24), a feature that gives the system the flexibility to properly choose the number of recording channels according to the specific experiment. The communication with a remote PC can be handled through Bluetooth and/or USB. All the device activities are managed by the low-power microcontroller (MSP430F5529). The system is equipped with a 3.7 V–200 mAh battery that gives an estimated run-time of about 28 h.

**Figure 2:**
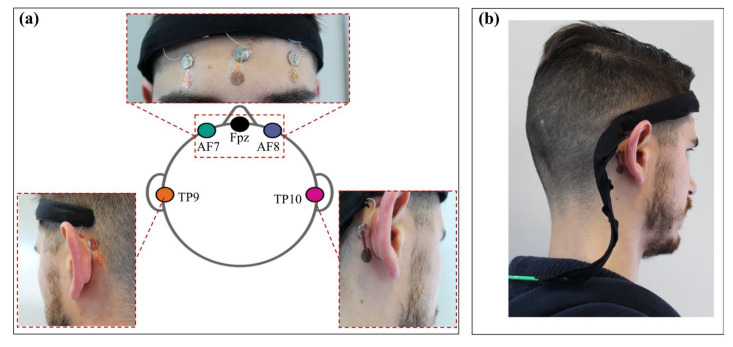
(**a**) Tattoo-electrodes are positioned following the 10–10 system, where the electrode in position Fpz is used as reference. The high conformability ensures a really good and high-quality contact with the skin, even in complex positions such as the mastoid process (electrode positions TP9 and TP10). (**b**) Rear view of the headset with the connector. The custom PCB is positioned on the back of the subject.

EXPERIMENTAL SETUP. With the aim to maximize the portability and the comfort of the wearable acquisition system, a low number of dry and unobtrusive tattoo electrodes placed according to the 10–10 standard was chosen. The system was validated in a standard resting-state experiment where eyes-closed and eyes-open conditions were contrasted, and the resulting performance was compared to that of the commercial wearable system MUSE headset (InteraXon Inc., Toronto, ON, Canada) [37,38,39]. To mimic the configuration of this commercial system, five electrodes were positioned on the forehead and on the mastoid, respectively, on AF7, Fpz, AF8, TP9 and TP10, as shown in Figure 2a. The electrode on Fpz was used as a reference electrode and the other active electrodes were measured with respect to it, in a unipolar configuration. The chosen placement of the electrodes allows the detection of the alpha waves to be maximized, especially in the occipital region, and the beta waves, on the frontal lobe, according to physiology [40]. For both systems, the EEG traces were recorded from a single subject during two minutes of eyes-closed and eyes-open resting-state conditions. As low-noise interconnections, 0.3 mm diameter micro coaxial cables from Alpha Wire were employed. In this way, due to the shielding, it was possible to reduce the power line interference, while still having flexible wires that could be easily integrated into a low-cost commercial headband. Each wire ends with a thin metal contact (1 cm in diameter in this preliminary version of the system), which is positioned on the skin using a small piece of non-conductive adhesive tape. The electrodes were simply placed face down (while still on the paper) with the recording area on the skin and the non-passivated final part on the metal contact. As previously described, after the removal of the paper the electrodes were able to conform to the skin without the need of any conductive adhesive layer.

The obtained flexible and lightweight structure allows an easy adaptation to the subject’s head, thus guaranteeing comfort and portability. The headset is physically connected to the PCB using a standard multipin connector placed at the end of a custom cable shown in Figure 2b. The communication between the device and the PC was handled using a custom MATLAB^®^ interface. Data were sampled at 250 sps (256 sps was the frequency rate for the MUSE headset) and elaborated offline using EEGLAB toolbox (version v2022.0) [41].

After the experimental session, the EEG signals (both those acquired using our system and those acquired with the Muse) were first band-pass filtered in the frequency range 1–40 Hz in order to discard the low-frequency drift and at the same time limit as much as possible the contamination of the signal due to muscle activity [42]. All the traces were successively inspected to remove residual artefactual segments.

## 3. Results and Discussion

**Figure 3:**
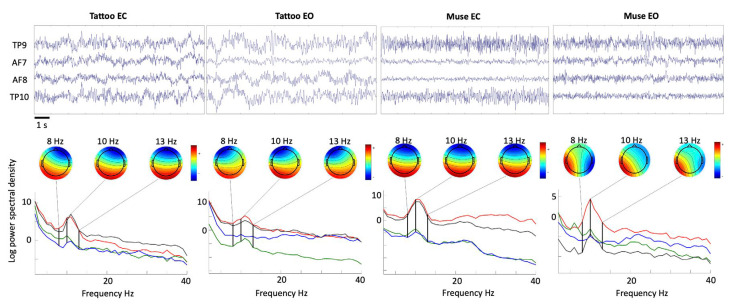
Exemplifying time-course of the EEG traces, channel spectra and maps for the two different acquisition systems and two different experimental conditions. EC and EO, respectively, refer to resting-state eyes-closed and eyes-open conditions. Each line represents a different channel, namely TP9 (red), TP10 (black), AF7 (green), AF8 (blue).

Figure 3 represents an exemplifying time-course of the EEG traces, channel spectra and maps for the two different acquisition systems in the two different experimental conditions.

Since the reference channel for the MUSE is located in correspondence to the Fpz position (which is not far from the frontal channels) and it has been shown that this choice may lead to asymmetric estimates compared with those evaluated using conventional montages [37], the frontal channel AF7 and AF8 were re-referenced to the TP9/TP10 mastoid electrodes. In order to evaluate if the recorded neural activity and the derived spectral measures obtained with the proposed system are reliable and able to capture the differences between the two experimental conditions (namely, eyes-closed and eyes-open resting-state), we tested whether these differences were comparable to those derived with the MUSE headset. The power spectral density (PSD) was computed using the pwelch function in MATLAB R2021b (The MathWorks Inc., Natick, MA, USA) for each of the frontal EEG channels separately and, successively, the average between the two channels was computed and used for the statistical analysis. The analysis was performed using, for each system and each condition, eighteen EEG segments (epochs) free of artifacts of 5 s extracted from the filtered traces [43]. The statistical analysis was conducted with JASP software (version 0.16.2—Apple Silicon) and performed using a paired nonparametric Wilcoxon signed rank test. The summary of the inference statistics, together with significance (*p* value threshold was set to 0.05) and effect size (rank-biserial correlation), are reported in Table 2 for the two systems separately.

**Figure 4:**
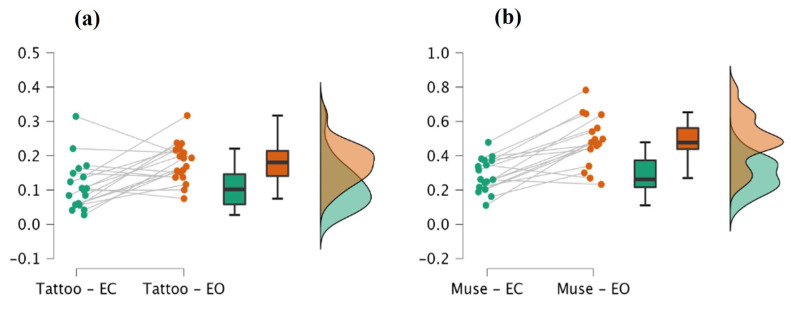
Raincloud plots for tattoo-based EEG system (**a**) and the Muse headset (**b**), with the underlying distributions of the relative power spectral density computed for the alpha frequency band.

As reported in Table 2, the results show an increase in the relative power spectral density during the eyes-open resting state conditions when compared to the eyes-closed conditions for both the proposed system (z = −2.286, *p* = 0.021) and the Muse headset (z = −3.385, *p* < 0.001). A slightly higher effect size for the Muse headset (−0.935) compared to the proposed system (−0.614) was observed. However, as expected, the two effects have the same direction. In Figure 4, we have reported the raincloud plots which show the underlying distributions of the relative power spectral density values for the two systems when contrasting the two conditions (namely, eyes-closed and eyes-open resting state). These results clearly show that the proposed system is reliable in capturing the differences between the two tested experimental conditions since it does not differ from the Muse headset, a commercial EEG system that was previously validated using a 64-channel research-grade EEG system [37]. The same results were obtained on two more healthy subjects as reported in the Supplementary Information (Figure S1), thus showing good results in terms of reliability. In order to evaluate the overall performance of our system, the same protocol was repeated on a healthy subject using standard cup electrodes, as shown in Figure S2 of the Supplementary Information.

In summary, the reported findings suggest that our system is able to detect the same direction of the effect observed using a commercial wearable EEG system in a well-known and very common experimental paradigm and that the magnitude of this effect is quite similar between the two approaches. This outcome is of particular relevance since it refers to EEG features, such as the spectral measures of frontal areas in alpha band, that have been shown to be of clinical and research relevance in many practical applications. Nevertheless, this work, for the EEG related part, has several limitations that need to be properly addressed in a follow-up study. The first limitation concerns the issue related to the small number of subjects. However, it is important to highlight that the statistical approach was adapted to this specific case and there is no reason to expect relevant differences among different subjects [44]. A second limitation concerns the investigation of a single experimental paradigm (i.e., resting state), although this is a standard and common procedure in EEG analysis. In the future, it would be interesting to investigate how the proposed system may respond to different experimental procedures in a multi-session and multi-subject study.

## 4. Conclusions

In this paper, a novel wearable system for the detection of EEG signals is presented. In particular, the system comprises a custom headband specifically designed to conveniently interface ultra-thin, dry tattoo electrodes, and a wearable, low-power electronic for the EEG signal acquisition. The system has been tested in a standard resting-state experiment and compared to a commercial EEG headset (MUSE— InteraXon Inc., Toronto, ON, Canada) showing consistent results in terms of discrimination between two different conditions (eyes closed and eyes open). Although preliminary, the obtained results showed the potential in terms of portability and comfort of wearable EEG systems based on unconventional electrodes. Indeed, the proposed solution represents an interesting example of an integrated system fully compatible with unobtrusive ultra-conformable electrodes of different kinds, thus paving the way to the development of a new generation of patient-centered EEG portable devices for applications such as non-invasive brain-computer-interfaces for prosthetics control and clinical neurophysiological studies.

## Figures and Tables

**Table 1:** Comparison between different EEG systems based on bioelectronic interfaces.

EEG Recording Systems	N° of Recording Channels	Electrodes Placement	Brain Wave Detected/ERPs	Portability/Wearable Acquisition System	Electrodes	Electrodes Thickness [µm]
Our Work	4	AF7, Fpz, AF8, TP9, TP10	Alpha and Beta	Custom portable PCB	Tattoo dry electrodes	0.7–0.8
V. Goverdovsky et al. [12]	1	Ear	SSVEP ASSR	Bench amplifier	Cloth electrode	//
S. Debener et al. [13]	16	Ear	Alpha, Beta and ERPSs	SMARTING (mBraintrain) mobile EEG amplifier	Flexible printed electrodes	//
J. J. S. Norton et al. [28]	1	Ear	P300 ERP	Bench amplifier	Tattoo dry electrodes	3
L. M. Ferrari et al. [29]	1	CZ-OZ T7-CZ	Alpha N100	Bench amplifier	Tattoo dry electrodes	1.5
H. L. Peng et al. [30]	1	FP1	N100	Bench amplifier	Dry electrodes	1200
S.Shustak et al. [31]	4	F7, F8	Alpha, k-complexes and spindles	portable PCB	Dry electrodes	//

**Table 2:** Summary and inference statistics for the two systems when comparing the two experimental conditions.

Wilcoxon Signed Rank Test					
Measure 1	Measure 2	W	z	*p*	Rank-Biserial Correlation
Tattoo-EC	Tattoo-EO	33.000	−2.286	0.021	−0.614
Muse-EC	Muse-EO	5.000	−3.385	<0.001	−0.935

## Data Availability

The data that support the findings of this study are available from the corresponding authors upon request.

## Supplementary Materials

The following supporting information can be downloaded at: https://www.mdpi.com/article/10.3390/s23020766/s1, Figure S1: Exemplifying time-course of the EEG traces and channel spectra and maps for the two different acquisition systems and two different experimental conditions (resting-state eyes-closed—EC—and resting state eyes-open—EO), recorded from subject 2 (a) and 3 (b). Figure S2: Time-course of the EEG traces and channel spectra and maps for the two different acquisition systems and two different experimental conditions (resting-state eyes-closed—EC—and resting state eyes-open—EO), recorded using commercial cup electrodes.
