# Supplementary material for: Wearable System Based on Ultra-Thin Parylene C Tattoo Electrodes for EEG Recording
Source: Sensors (Basel). 2023 Jan 9;23(2):766. doi: 10.3390/s23020766 (PMC9861766; doi:10.3390/s23020766)
Supplement: Supplementary file 1 [file sensors-23-00766-s001.zip › sensors-2115293-supplementary.pdf]

Wearable System Based on Ultra-Thin Parylene C Tattoo Electrodes for EEG Recording

Antonello Mascia [1,†], Riccardo Collu [1,†], Andrea Spanu [2,*], Matteo Fraschini [1,*], Massimo Barbaro [1] and Piero Cosseddu [1]

[1] Department of Electrical and Electronics Engineering, University of Cagliari, Piazza D'Armi, 09123 Cagliari, Italy
[2] Scuola Universitaria Superiore IUSS, Palazzo del Broletto Piazza della Vittoria15, 27100 Pavia, Italy
* Correspondence: andrea.spanu@iusspavia.it (A.S.); fraschin@unica.it (M.F.)
† These authors contributed equally to this work.

EXPERIMENTAL RESULTS AND DISCUSSION

Measurements reported in the experimental results and discussion session were reproduced on two different healthy male subjects. The results are reported in S1 and, although still preliminary, highlight a good reproducibility and reliability of the proposed system.

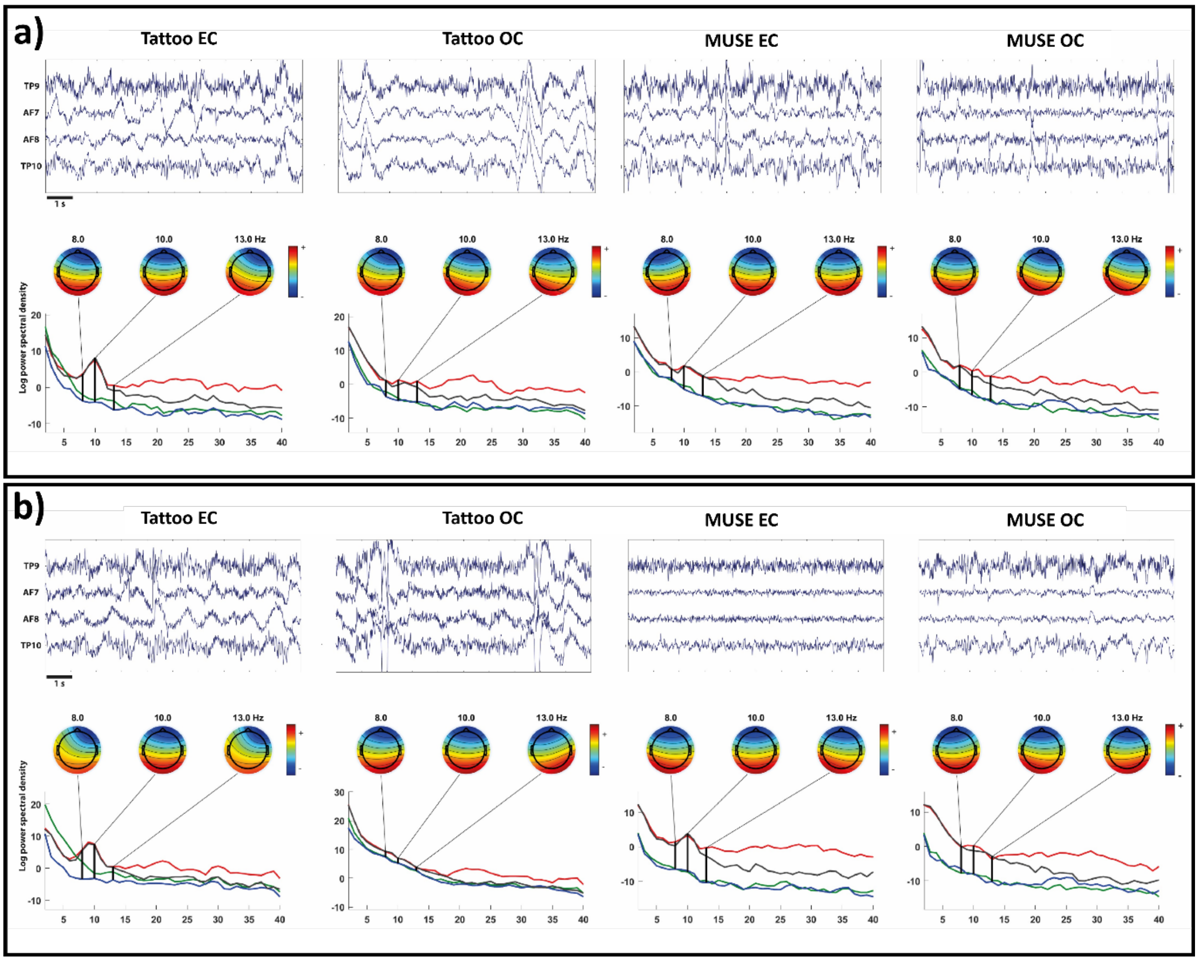

Figure S1. Exemplifying time-course of the EEG traces and channel spectra and maps for the two different acquisition systems and two different experimental conditions (resting-state eyes-closed – EC – and resting state eyes-open - EO), recorded from subject 2 (a) and 3 (b). Each line represents a different channel, namely TP9 (red), TP10 (black), AF7 (green), AF8 (blue).

The same experimental protocol previously reported in the experimental results and discussion session was applied on a healthy male subject using commercial cup electrodes as reported in Figure S2.

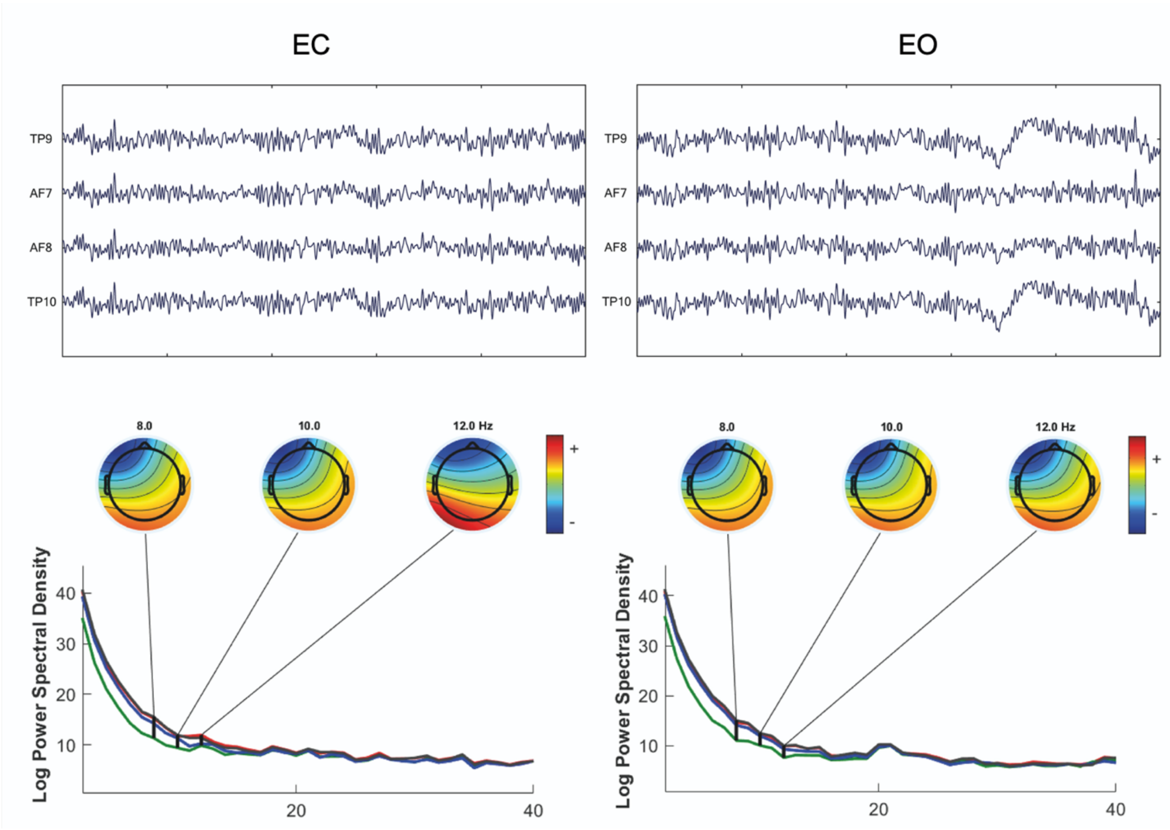

Figure S2. Time-course of the EEG traces and channel spectra and maps for the two different acquisition systems and two different experimental conditions (resting-state eyes-closed – EC – and resting state eyes-open – EO), recorded using commercial cup electrodes. Each line represents a different channel, namely TP9 (red), TP10 (black), AF7 (green), AF8 (blue).
